# Sensors and Clinical Mastitis—The Quest for the Perfect Alert

**DOI:** 10.3390/s100907991

**Published:** 2010-08-27

**Authors:** Henk Hogeveen, Claudia Kamphuis, Wilma Steeneveld, Herman Mollenhorst

**Affiliations:** 1 Chair group-Business Economics, Wageningen University, Hollandseweg 1, 6706 KN Wageningen, Netherlands; 2 Department of Farm Animal Health, Faculty of Veterinary Medicine, Utrecht University, Yalelaan 7, 3584 CL Utrecht, Netherlands; E-Mails: c.kamphuis@uu.nl (C.K.); w.steeneveld@uu.nl (W.S.); h.mollenhorst@uu.nl (H.M.)

**Keywords:** clinical mastitis, sensors, electrical conductivity, algorithms

## Abstract

When cows on dairy farms are milked with an automatic milking system or in high capacity milking parlors, clinical mastitis (CM) cannot be adequately detected without sensors. The objective of this paper is to describe the performance demands of sensor systems to detect CM and evaluats the current performance of these sensor systems. Several detection models based on different sensors were studied in the past. When evaluating these models, three factors are important: performance (in terms of sensitivity and specificity), the time window and the similarity of the study data with real farm data. A CM detection system should offer at least a sensitivity of 80% and a specificity of 99%. The time window should not be longer than 48 hours and study circumstances should be as similar to practical farm circumstances as possible. The study design should comprise more than one farm for data collection. Since 1992, 16 peer-reviewed papers have been published with a description and evaluation of CM detection models. There is a large variation in the use of sensors and algorithms. All this makes these results not very comparable. There is a also large difference in performance between the detection models and also a large variation in time windows used and little similarity between study data. Therefore, it is difficult to compare the overall performance of the different CM detection models. The sensitivity and specificity found in the different studies could, for a large part, be explained in differences in the used time window. None of the described studies satisfied the demands for CM detection models.

## 1. Introduction

There are many aspects to milk quality. Mastitis is associated with two of the milk quality aspects that are used in most dairy producing countries: somatic cell count (SCC) and, if mastitis is clinical, visibly abnormal milk (in the remaining part of this article referred to as abnormal milk). In most dairy systems it is assumed that the farmer, informed by the official organizations in his country, has the responsibility to deliver milk of sufficient quality. In order to deliver milk with a low SCC, attention should be given to an adequate detection and prevention of mastitis. Efficient detection of clinical mastitis (CM) is therefore important. A well-established method to detect CM is to strip before milking and check the foremilk for abnormalities. Discarding of abnormal milk is part of the EU Milk Hygiene Directive (EC/92/46). Milk from diseased cows or milk that is visually abnormal should not be delivered. Discarding of abnormal milk is also mandatory in the USA, according to the Grade “A” Pasteurized Milk Ordinance. Checking of foremilk is thus important to detect CM and to meet the requirements of milk quality legislation.

After development of individual animal identification [1], applications have been sought for this technique. Individual feeding, individual milking and automated detection of events of interest for the farmer are all applications following the development of individual animal identification. Because of the costs of mastitis [2,3], this disease has been the first focus of sensor developments in the dairy sector. From the mid-eighties, work has been carried out in order to automate the detection of mastitis by means of sensors. During milking, abnormal milk was detected using visual observations [4,5]. When detection of CM is carried out automatically, the task of the milker becomes easier and the capacity of milking parlors can be increased. Although sensors for detection of mastitis became commercially available in the beginning of the nineties, they never were applied on a large scale. Because of the fact that with automatic milking no milker is present at the time of milking, the need for sensors to detect CM and abnormal milk was high when automatic milking systems were commercially introduced in 1992. In the last years, the number of farmers with an automatic milking system has increased considerably. In the Netherlands, currently approximately 10% of the dairy farmers are milking their cows with an automatic milking system. Moreover, because of the number of milking clusters in an automatic milking system is much lower than in a comparable milking parlor, the costs of application of sensors is also lower in an automatic milking system. Therefore, interest in the application of sensors to detect mastitis and abnormal milk has been increasing considerably in recent years (e.g., [6]).

The performance of the sensors currently used in practice (mostly based on electrical conductivity of milk), should be improved considerably. Especially the large number of false positive alerts is a concern for many dairy farmers. There is a strong need for performance improvements. There are two major routes through which this can be done: improvement of sensors and improvement of detection models that translate the sensor data into information for the herdsman. This paper describes the demands for performance of sensor systems to detect CM and evaluates the current performance of these systems. Therefore, this paper first starts with a section on performance demands, followed by a section on different types of sensors, different algorithmic approaches and the possibilities of combining sensors with other sensors and knowledge from other sources.

## 2. Demands for Automatic Detection of Clinical Mastitis

Table 1 summarizes the study characteristics and results from previously conducted studies on CM detection models using sensory data. All sensors listed in Table 1, except the SCC sensor [7,8] are in-line sensors. The SCC sensor is an on-line sensor, where a small amount of milk is separated from the milk tube and led through the sensor. Table 1 only includes results from peer-reviewed studies that used sensors for the development of CM detection models. Therefore, some recent studies are not included in this table as these studies used cow information [9] or laboratory determined l-lactate dehydrogenase (LDH) [10–12], or they were not peer-reviewed [13]. The number of studies included in Table 1 makes clear that a lot of research on automated CM detection models has been done. It also makes clear that comparing results between the different studies is not valuable, as the characteristics between the studies differ too much. In this section, the demands for a detection model for CM will be described and discussed in relation with results of published studies.

### 2.1. Detection performance

#### 2.1.1. Measuring performance

Sensors for detection of mastitis and/or abnormal milk can be seen as diagnostic tests, which can be characterized by epidemiological parameters. It is very important that the event of interest is clearly defined; especially because the demands for a test might differ for the event of interest. For instance, detection of (visual) abnormal milk is done for other purposes than detection of CM. In the following sections these events will be described in the light of demand for performance of sensors.

When evaluating a sensor, this is done in an experiment where the alerts given by the sensor are compared with the occurrence of an event in reality (gold standard). The outcomes of such an experiment can be classified as follows:

- Number of observations where the event occurs with an alert (*TruePosCount*)
- Number of observations where the event occurs without an alert (*FalseNegCount*)
- Number of observations where the event does not occur with an alert (*FalsePosCount*)
- Number of observations where the event does not occur without an alert (*TrueNegCount*)

Using these basic classifications, the performance of a sensor can be evaluated as follows. The two most important parameters are sensitivity and specificity:

$$\begin{array}{l} \mathrm{Sensitivity (\%) = 100 \times TruePosCount/(TruePosCount + FalseNegCount)}\\ \mathrm{Specificity (\%) = 100 \times TrueNegCount/(FalsePosCount + TrueNegCount)} \end{array}$$

The sensitivity refers to the probability that the event of interest (e.g., a milking with CM) will be classified as such (positive test result; alert). The specificity refers to the probability that when the event of interest does not occur (e.g., a milking without CM) will be classified as normal (negative test result; no alert). Sensitivity and specificity are interdependent. If the threshold of a test is increased, the number of positive outcomes and thus the sensitivity will decrease. On the other hand, the specificity will increase. Therefore, thresholds have to be set in such a way that the performance of a sensor in terms of sensitivity and specificity is optimized.

The sensitivity and specificity of a test are independent of the occurrence of the events (prevalence). For a practical evaluation of sensors, the prevalence of the event of interest is important. The prevalence of CM is very low. Prevalence of CM is approximately 0.04%, equivalent to four cases per 10,000 milkings [28]. The prevalence of subclinical mastitis is, depending on the definition of subclinical mastitis, approximately 100–5,000 cases per 10,000 milkings. This low prevalence, especially for CM, will have effects on the interpretation of sensor data. The farmer does not see the gold standard, but sees alerts. To evaluate sensors to detect mastitis or abnormal milk from a farmer’s point of view, the following two definitions are proposed [27]:

$$\begin{array}{l} \mathrm{Success Rate = TruePosCount/(TruePosCount + FalsePosCount)}\\ \mathrm{False Alert Rate = 1,000 \times FalsePosCount/TotalCowMilkings} \end{array}$$

Note that we have deliberately avoided the commonly employed terms true positive, false positive, true negative and false negative because of inconsistency in their usage. For instance, consider the following three definitions of a true positive as: ‘a case of mastitis where one or more alerts are given’ [17]; ‘an alert during a mastitis period’ [19]; and ‘an alert on the day of observation’ [22]. Each is capable of generating a different true positive list from the same basic data. Success rate could be a more useful statistic, giving a more direct measure of the proportion of alerts that are likely to be correct. Success rate is a synonym for the positive predictive value. A downside of success rate is that it is not an ‘absolute’ statistic. Thus, the success rate will vary with the prevalence of the condition being monitored. This downside can be avoided by calculating the total number of false alerts over a given number of cow-milkings, e.g., the total number of false alerts per 1,000 cow-milkings. This expression of the ‘false alert rate’ would be a simple, practical and comprehensible measure to which farmers will readily relate. The false alert rate is essentially 10 × (100% − specificity) per 1,000 cow-milkings. This approximation should always be close enough for practical purposes since in normal situations, the prevalence of mastitis or abnormal milk is very low relative to the total number of cow milkings.

#### 2.1.2. Demand for performance to detect clinical mastitis

The primary goal for on-line detection of CM is to be able to cure the diseased cow. After an alert signal, the herdsman will check the cow first to confirm the mastitis before deciding on treatment. The advantage of using a sensor system to detect CM is the management by exception principle. Only those cows requiring attention will get it. Because a mastitis case will be confirmed first, a slightly higher false alert rate will not be a problem. The only costs incurred arise from the check made by the herdsman. Alerts can be given directly in the milking parlor, decreasing the time that is needed to milk one cow. Currently, sensors to detect CM are mostly applied in automatic milking systems. In these systems, alerts are placed on a list. The herdsman checks this list regularly and has to checks the cows on it. Costs involved with checking are higher, because it requires more (annoying) labor to find the cow, fetch her and check her somewhere in the barn. For use with an automatic milking system, therefore, the false alert rate should be lower. It is important that as many cows with CM as possible (preferably all) will be identified, requiring a high sensitivity. From a welfare point of view, at least cows with severe CM (grave systemic and local symptoms) must be detected. Time windows for CM should be short. A cow with CM should be treated soon after the onset of clinical signs. When a cow receives an alert when there are no clinical signs yet, the farmer will check the cow and determine that it was a false alert and do nothing, while in fact the cow would become clinical soon. In the next section, this will be discussed further.

The International Standard ISO/FDIS 20966 (Automatic milking installations—requirements and testing) of the International Standard Organization includes an Annex, attempting to deal with methods of detecting abnormal milk and interpretation of test results. This annex describes a minimum sensitivity of 80%, combined with a specificity better than 99% (∼false alert rate smaller than 10 per 1,000 milkings). These recommendations are, however, still under discussion [29]. Looking at Table 1, shows a very large variation in results. Sensitivities range from 47% [8] to 100% [18,19] and specificities range from 69% [16] to 99.8% [19].

### 2.2. Time window of detection

**Figure 1.**
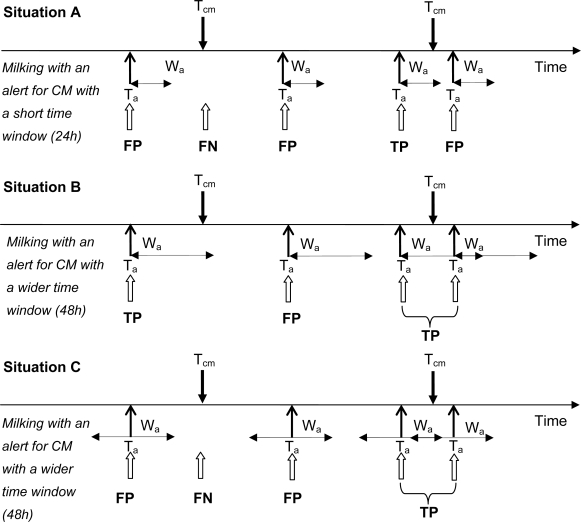
Applying different time windows (24 h, situation A; 48 h situation B; 24 h before and after an alert, situation C) to alerts (black arrows pointing up) for clinical mastitis (CM; black arrows pointing down) and its effect on the false positive (FP), false negative (FN), true positive (TP), and true negative (TN) alerts (white arrows pointing up). An FP alert occurs when an alert for CM (T_a_) extended with a time window (W_a_) has no observation of CM (T_cm_) falling into that time window. An FN alert occurs when there is a T_cm_ without any overlapping W_a_. A TP alert occurs when there is a T_a_ with an extended time window W_a_, in which a T_cm_ falls. When two W_a_ overlap each other (see situation B and C) these alerts are labeled as one TP (in both situations B and C), FP, or FN.

A basic evaluation method is to compare alerts with events at the same moment. For instance, for 1,000 milkings visual evaluation of normality of milk is carried out. These data are used as the gold standard. An event is defined as the occurrence of abnormal milk. When, during the same milking the sensor is used and alerts are generated, the counts of events in relation to alerts can be evaluated as above [8,15]. However, when the timing of observations of events and alerts does not occur at the same moment, time windows have to be used to combine those observations (e.g., [25]). Especially when the time between observations is variable, the use of a time window becomes more complex [27]. Observations of CM as well as alerts for CM by a detection model are points in time. To both events time windows can be applied, although in most conducted studies time windows are only applied to an alert for CM by a detection model. The time window is then used to formulate a time episode in which an alert by the CM detection model is a valid one. Figure 1 explains the application of time windows to CM alerts by a detection model graphically, and its effect on the false positive, false negative, true positive, and true negative alerts in a setting with an automatic milking system. When milking with an automatic milking system, dairy producers can detect CM visually at any time of the day, and mostly these times do not coincide with a milking (this in contrast to farmers that milk conventionally in a milking parlor). The CM detection model however, alerts at the time a cow is being milked by the automatic milking system. Figure 1 depicts three situations; the first uses a short time window in which an alert by the detection model is valid (24 h; situation A), the second uses a wider time window of 48h (Situation B), and the latter uses a total time window of 48 h but split into 24 h before and after an alert for CM (Situation C). These time windows are example time windows for the detection of CM, but they show clearly the effect of using different lengths of time windows, as well as the effect of applying time windows only after an alert or both before and after and alert. In situation A, the detection model has three false positive alerts, there is just one false positive alert in situation B, and two in situation C. Figure 1 explains visually that when time windows become too small, too many true CM cases will be missed (situation A has one true positive alert, situation B has two true positive alerts). When it is too wide, more CM cases will be accounted for as true positive alerts, but the model will lose its practical ability as dairy producers will perceive these alerts as false positive (as no signs of mastitis are visible yet). Also, when time windows are applied before and after a CM alert (situation C), it is possible that an alert given 24 h after a CM observation may be perceived as too late by dairy producers.

If it is no problem when an alert is given up to fourteen days after the onset of an event, an alert will regarded as true positive, when it is given within fourteen days of the onset of the event. This knowledge can be used calculating the TruePosCount and TrueNegCount. In general, performance of sensors will improve when larger time windows are used. When the used time window during evaluation does not match practice, the actual use of sensors might lead to disappointment of the farmer. Therefore, time windows should be considered carefully.

**Figure 2.**
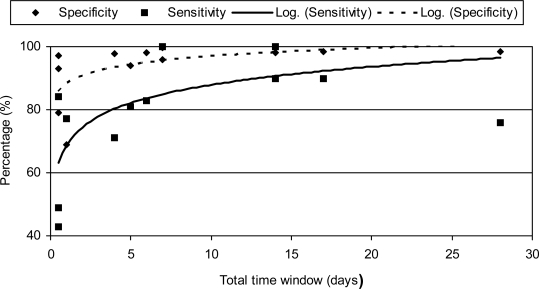
Sensitivities and specificities (y-axis) of various studies, plotted against the used time windows (x-axis). Given are the observations and a logarithmic trendline.

In past research on automated CM detection models, time windows have been widely varied; from 0 days to 17 days (Table 1). With very wide time windows, e.g., a time window of 17 days used by De Mol *et al*. [17], usefulness of the detection model will be low. What the right time windows would be for a farm with an automatic milking system is arbitrary. However, for the detection of CM, it should not be too wide as it is of great importance that CM be detected shortly after clinical signs appear in order to eliminate the disease and to prevent recurrence [30]. An alert that is given before clinical signs can have advantages, because it can be seen as an early detection and treatment will have a higher efficiency [31]. However, the specificity of such an alert must be very high to prevent the unnecessary use of antibiotics [32] Also when a model is used to divert abnormal milk automatically, the applied time window should be extremely narrow, as it applies to the specific milking that shows abnormalities. Diversion of abnormal milk should be preferable at the first milking, but depending on severity could be at a following milking. Another interesting observation is the relation between used time window of evaluation and performance of the system. With a longer time window, the performance increases (Figure 2).

### 2.3. Similarity of study population with the real application

A CM detection model should be evaluated on a population with a large similarity with the situation of the actual application of the system. This may seem logical at first, but most studies reporting on CM detection models lack this essential requirement and their application with field data might be disappointing. Reasons for not fulfilling this requirement are: (1) the use of a small array of data, (2) definition of CM and non-CM milkings and (3) performance with missing data. In the next paragraphs these reasons will be worked out further.

Herds differ from each other with respect to the CM situation. That means that if a detection model is based on one herd, the performance of this model in other herds might be disappointing. Moreover, if the herd used to collect data to build a model is a research herd, the data might be better than in practice and consequently, the model might perform less in the field than in the research. Also the use of a herd with a high prevalence of CM for data collection may be representative for a specific range of herds in practice (those where there are problems with detecting CM or with diversion of abnormal milk, but data from such a high prevalence herd is not representative for the whole population of dairy farmers. Most published studies were conducted with data from one (research) herd, and included a small number of CM cases (Table 1). A lot of studies did develop and validate a model using different data sets, but data for both sets still came from the same herd (e.g., [26]). These approaches of using one herd for training and testing may result in a model that detects CM at high levels of sensitivity and specificity, but this does not predict the performance on data of a new farm. The risk of including a small number of CM cases (e.g., 13 CM cases were used by Nielen *et al*. [16]) is that these cases may not represent all variation in CM characteristics (in terms of clinical signs and in sensor measurement patterns), causing a decrease in performance when the model is applied on other herds.

It is not always easy to distinguish CM milkings from non-CM milkings. For example, cows may be challenged by pathogens invading the udder, but this infection may have not reached a clinical level (yet). These pathogens cause changes in milk composition that may be detected by sensors, but not yet by the human eye. It is therefore, sometimes difficult to distinguish milkings with CM from milkings without CM. To generate contrast, detection models can be build using clear CM milkings and clear non-CM milkings by adding strict inclusion criteria. However, in practice, these not very clear non-CM milkings are part of the milkings that have to be evaluated. When a CM detection model alerts for milkings that do not have CM, dairy producers will perceive these alerts as false positive ones and they will judge the CM detection model as being inaccurate. Some studies did use strict definitions for healthy cows or quarters and those that suffered from CM, where SCC, bacteriological culturing, and visual observations are used to define ‘healthy’ and ‘diseased’. This strict definition has been used for cases and non-cases in datasets used for training and for datasets used for validation (e.g., [15,21]). For example, Friggens *et al*. [11] introduced an interesting new approach of presenting a mastitis risk, rather than presenting a binary classification. The model showed high levels of sensitivity and specificity, but performance was based on a validation set that included only highly selected cases and non-cases for CM. However, as a consequence of their strict selection criteria, cows and quarters with a less clear health status were excluded. This does not coincide with daily practice on a dairy farm.

In practice, it often occurs that data are missing. It is therefore better that detection models are capable of detecting CM in a situation with missing data. When during model building missing data are left out, the performance of the final detection model might look better than in reality. Some of the published studies excluded complete records with sensor measurement errors or missing data (e.g., [20]) because the used methodology to develop a CM model was not able to deal with such data. However, real farm sensor data are noisy, due to missing values and the need for calibrations of sensors to guarantee a proper functioning and monitoring. Therefore, a CM detection model should be able to deal with these noisy data as well.

## 3. Sensors to Detect Clinical Mastitis

Because of the physiological changes in the udder, intramammary infections lead to major alterations in the composition of milk [33]. Many sensors have been proposed that measure one or more of the inflammation parameters. Recently, two reviews have been published on sensors and udder health [6,34]. In this section a summary of the most important sensors where CM detection models have been built with will be given.

### 3.1. Electrical conductivity

Electrical conductivity (EC) is a measure of the resistance of a particular material to an electric current. In milk, ions present are the main component conducting electricity. Active and passive transport systems in the secretory cells of the mammary gland keep the sodium-potassium ratio in the milk approximately 1:3, whereas it is 30:1 in extracellular fluid or blood. The chloride concentration in milk is much lower than in blood. The mammary ducts are impermeable to ions. Mastitis leads to a change in blood capillary permeability, destruction of tight junctions and the destruction of the active ion-pumping systems. As a result the ion concentrations in milk change. Since milk is iso-osmotic with blood, the secretory cells of the mammary gland will stabilize the osmotic pressure leading to a change in EC [35]. This change in EC can be used as indicator for CM. However, the EC is also affected by other reasons than mastitis [36] such as temperature, the fat content of milk and milk fraction.

Because the principle of measuring EC is relatively simple, sensors for measuring EC are commercially available for a number of years. Basically there are two types of systems available: (1) systems that measure the conductivity of the whole milk, located for instance in the electronic milk meter and (2) systems measuring the conductivity per udder quarter, located in the claw of the milking cluster (traditional milking systems) or in the long milk tube (automatic milking systems). Since mastitis is an event which occurs on udder quarter, EC measurements on quarter level give the possibility to compare udder quarters, thus increasing the test characteristics [8]. Most of the published studies do use EC as sensor (Table 1), with a large variation of performance results.

### 3.2. l-Lactate dehydrogenase

LDH is the result of one of the enzymatic reactions following mastitis. It is part of the glycolytic pathway, found in the cytoplasm of all cells and tissues in the body. LDH is a responsive indicator of mastitis as a result of the animal’s immune response against infection and changes in cellular membrane chemistry. LDH has a large potential for detection of CM [11]. A bio-sensor, using dry-stick technology has been evaluated [10] using simulated data and data from one research farm where milk samples were analyzed in a laboratory with good performance results. This sensor is commercially available [37], but performance of this system has not been systematically evaluated.

### 3.3. Color

A direct measure of the physical characteristics of abnormal milk (mostly due to CM) will most likely offer better detection results than a measurement of an indirect indicator of mastitis or abnormal milk. One of the visible aspects of milk is its color. A sensor for on-line color measurement is on the market. The principle of the sensor is based on the reflection of light generated by a LED. The whiter the milk, the more light is reflected. Three different wavelengths of light are measured by the sensor: red, green and blue. Recently a new version of a color sensor became available, which is based on light transmission and not on light reflection. The results of this sensor seem to be better than of the older version [13].

In a first study under laboratory circumstances, using homogenised quarter milk samples from eight cows with CM the potential to detect mastitis from color measurements was estimated. The milk samples of the suspected quarters of all eight cows with CM showed lower color values than homogenized milk [38]. In a detailed study on the predictive potential of EC and color measurements, it became clear that most information to distinguish udder quarters with abnormal milk and CM from other udder quarters could be found in EC measurements [7]. The potential of color measurements did add but not very much. This means that color sensors should always be used in combination with other sensors.

### 3.4. Somatic cell count

When the mammary gland becomes infected, a rapid influx of polymorphonuclear leukocytes leads to an increase of the SCC [39]. Rapid reliable measurement of SCC is carried out routinely in laboratories, and can be used to monitor udder health. Therefore, SCC is used as an important tool for the control of mastitis. Near infrared (NIR) has shown to be able to measure, amongst others, SCC in raw milk (e.g., [40]). A commercial available NIR analyzer has been described [41] but is not available on-line. Sensors that measure SCC on-line, based on the principles of the Californian mastitis test [42] or on staining a milk sample and count the actual number of cells optically [43] are commercially available on automatic milking systems today. The sensor based on Californian mastitis test principles utilizes the gel-formation process of this Californian mastitis test. The potential value of this sensor has been studied at the cow level in combination with EC. With thresholds set in such a way that the sensitivity of the detection model was 80%, on-line SCC measurement gave similar results as EC. When combining EC and SCC measurements, the performance of the detection model improved [23]. Recent data show that measuring SCC on quarter level gives better detection performance than measuring SCC on cow level [8]. The optical cell counter is based on a system that makes a picture of a milk sample that is mixed with a staining solution, and that counts the number of stained cells. The sensor has been tested recently, with a positive trend to identify cows with somatic cell counts of 200,000 cells/mL or more [43].

### 3.5. Homogeneity

CM is defined as a situation where a cow’s udder is infected leading to visual changes in the milk, the cow or both. Visual detection of CM by the farmer occurs through checking of the first few squirts of milk, before milking. When the milk is not white or homogeneous, the cow is suggested to have clinical mastitis. Homogeneity of milk can therefore be an interesting parameter for mastitis detection, for instance by image processing or diffusing wave spectroscopy [44,45]. A sensor to measure homogeneity of milk has been suggested [46], but no information is available on further developments on this specific type of sensor.

## 4. Algorithms

Sensors, however advanced they might be, deliver data. Many on-line sensors deliver a large amount of data. There are many measurements per milking, sometimes per udder quarter and during many milkings. These data by themself are not informative, and should be processed to generate information. A good algorithm is essential to optimize and convert the on-line sensor data into an interpretable value. Algorithms can make a huge difference in the performance of a sensor system. The simplest form of processing is to take the output value of a sensor and compare this output value with a threshold. However, most on-line sensors measure continuously and data have to be pre-processed to generate an end value that can be compared with the threshold. Moreover, for many sensors it is not the absolute level, but the relative level (relative to previous milkings or relative to other udder quarters) that is more related to CM. Moreover, data from different sensors can be used in combination in a CM detection model. Much work on algorithms to improve CM detection models is focused on data pre-processing. Before starting model building it is important to know the characteristics of algorithms and choose a suitable algorithm for the particular modelling purpose [47]

Described algorithms include the use of thresholds (e.g., [8]), moving averages (e.g., [14]), neural networks (e.g., [16]), multivariate regression models (e.g., [15,48]), fuzzy logic (e.g. [19,22,23]) and datamining (e.g., [7,25]). The principle of moving averages has been extended considerably by fitting time series models such as Kalman filters (e.g., [11,48]) or locally weighted polynomial regression (e.g., [49]).

In a series of studies, Cavero *et al*. [22,49,50] applied three different algorithms to data from the same research dairy farm, using equal definitions (a case was based on a combination of SCC and treatment for CM). The found sensitivities of these studies were 83, 88 and 79% respectively for fuzzy logic, locally weighted polynomial regression and neural networks with, respectively a specificity of 76, 67 and 61%. Although the performance of these methods do differ, these differences are not very large. Unfortunately, these three studies were carried out on datasets with different sizes, so a good comparison between the methodologies could not be made.

## 5. Combining Sensors and Other Information

Because mastitis is associated with many changes in the cow and milk, a combination of more than one sensor has been proven to be useful. The most used sensor, the one for EC, is often combined with measures of milk yield and milk temperature (Table 1). In general, the studies that use a combination of sensors have a better performance than studies that look at a single sensor. Some studies clearly proved that a combination of data from different sensors improves the detection performance (e.g., [8,23]). Moreover, data measured by a sensor can be summarized in a number of detection parameters [51].

A final method that is proposed to improve the detection performance of sensors, is the combination of sensor output with other, non-sensor information of the cows, such as lactation stage and mastitis history [10,52,53]. It is applied in one described system [10], but from that publication it was not clear what the addition of the non-sensor information added to the detection performance. Very recent data showed that, although cow information does predict the risk of CM and thus the prior probability of CM, these additional data did not add much to the detection performance of sensor systems [54]. Therefore, not too much must be expected from this combination of sensor data and other cow information.

## 6. Concluding Remarks

The use of sensors to improve milk quality has gained much attention lately. This is largely because of the demand of good performing sensors to be used in automatic milking systems. The most studied and applied sensor measures EC. Besides the development of automatic milking systems, there are new sensor developments, for instance the use of NIR, and measurement of SCC and LDH, that make interesting future improvements possible. Many ideas for bio-sensors to detect CM have been described [34], but are not available in practice (yet).

When evaluating a detection model based on a specific sensor or combination of sensors, it is very important to define the event that needs to be detected [32]. Moreover it is important to consider an appropriate time window and the final application of the detection model. For detection of CM a relatively short time interval (24–48 hours) should be used.

Performance of CM detection system should offer at least a sensitivity of 80% and a specificity of 99%. The time window should not be longer than 48 hours and study circumstances should be as similar to practical farm circumstances as possible. The study design should comprise more than one farm for data collection.

For future applications, the definition of mastitis might be reconsidered. Currently there is still a distinction between clinical and subclinical mastitis. With renewed interest and found efficiency of treating subclinical mastitis cases, it might be interesting to get away from a binary mastitis variable and to go to a continuous mastitis variable. A first step towards such a system of thinking has recently been made with the introduction of “Degree of infection” [55]. Currently we are at the beginning of a series of new developments in this area. The quest for a perfect detection of CM is only beginning.

## Figures and Tables

**Table 1.** Study characteristics of peer-reviewed published studies conducted from 1990 onwards that used sensor information (in- and online) for the detection of clinical mastitis. Characteristics included are the number of farms, definition of non-cases and cases and the number of them included in the study, sensors used, the applied methodology, the time window applied for classification, the sensitivity (SE), and the specificity (SP).

**Paper**	**Farms (n)**	**Definition non-cases (n)**	**Definition cases (n)**	**Sensors**	**Algorithm**	**Time window**	**SE (%)**	**SP (%)**
Maatje *et al*., 1992 [14]	1 research farm	Based on bacteriological culturing and SCC^1^ (200)	Clinical mastitis based on bacteriological culturing and SCC (25)	EC^2^	Moving average and threshold	14d	100	-
Nielen *et al*., 1995 [15]	1 research farm	Based on bacteriological culturing and SCC (25)	Clinical mastitis based on observing abnormal milk (31)	EC, milk yield, milk temperature	Artificial Neural Network	0d^3^	84.0	97.0
Nielen *et al*., 1995 [16]	1 research farm	Based on bacteriological culturing and SCC (17 for training; 13 for testing)	Clinical mastitis based on observing abnormal milk or signs of inflammation (13 for training; 13 for testing)	EC, milk yield, milk temperature	Artificial Neural Network	1d	77.0	69.0
De Mol *et al*., 1997 [17]	2 research farms	-- (6,495 milkings)	Clinical mastitis based on clinical signs (52 cases)	EC, milk yield, milk temperature	Time-series with Kalman filter	17d	90^4^	98.2^5^
De Mol and Ouweltjes, 2001 [18] ^6,7^	1 research farm	Based on never having clinical mastitis, bacteriological results, and SCC (29,033 milkings)	Clinical mastitis based on clinical signs (48 cases)	EC, milk yield, milk temperature	Time-series with Kalman filter	7d	100^4^	95.1^5^
De Mol and Woldt, 2001 [19]	1 research farm	Based on never having clinical mastitis, bacteriological results, and SCC (29,033 milkings)	Clinical mastitis based on clinical signs (48 cases)	EC, milk yield, milk temperature	Fuzzy Logic	7d	100^8^	99.8
De Mol *et al*., 2001 [20] ^7^	4 semi-research farms	Based on not having CM in the collection period, SCC and times milked (299,842 milkings)	Clinical mastitis based on visual observation (95 cases)	EC, milk yield, milk temperature	Time-series with Kalman filter	4d	67^4^	97.9^5^
Norberg *et al*., 2004 [21]	1 research farm	Based on bacteriological culturing and having no treatment for clinical mastitis by veterinarian (1,353)	Clinical mastitis based on treatment by veterinarian after observing clinical signs by staff members (275)	EC	Discriminant function analysis	0d^3^	47.9	91.9
Cavero *et al*., 2006 [22]	1 research farm	Based on not being treated for clinical mastitis (109,690 healthy days for training; 51,588 healthy days for testing)	Clinical mastitis based on treatment (651 days of mastitis for training; 348 days of mastitis for testing)	EC, milk yield, milk flow	Fuzzy logic	5d Day of treatment, plus 2d prior and 2d after treatment	92.9	93.9
Kamphuis *et al*., 2008 [23]	1 research farm	Based on milkings without treatment records (27,699 cow milkings)	Treated cases of clinical mastitis (18 cow milkings)	EC, SCC	Fuzzy Logic	2d for alert by model, 1d for observation	80	99.2^10^
Claycomb *et al*., 2009 [24]	1 for training 1 for testing	--	Clinical mastitis as clots on filter (23 in test set)	EC	Threshold	4d/2d	83	99.8
Mollenhorst *et al*., 2010 [8]	3 commercial farms	Based on visual normal milk (3,172 quarter milkings)	Clinical mastitis based on visual observation of abnormal milk (19 quarter milkings)	EC, SCC	Threshold	0d^3^	47.4	99.0
Kamphuis *et al*., 2010 [25]	6 commercial farms	Based on visual checks of farmers or on random selection (3,000 quarter milkings)	Based on visual observation by farmers (97 quarter milkings)	EC, color, milk yield	Decision-tree induction	<1d	32.0	98.7
Kamphuis *et al*., 2010 [7]	9 commercial farms	Training: cases checked for clinical mastitis and SCC (24,960 quarter milkings). Testing: no observation of CM and without a 2-week range of a CM case (50,000 quarter milkings)	Based on visual observation by farmers (243 for training; 105 for testing)	EC, color, milk yield	Decision-tree induction	<1d	40.0	99.0
Sun *et al*., 2010 [26]	1 research farm	Based on SCC and not being treated for clinical mastitis (3,235 quarter milkings)	Clinical mastitis based on visual observation by farm staff or SCC (895 quarter milkings)	EC, milk yield	Artifical Neural Network	0d^3^	86.9	91.4

1 Somatic Cell Count.

2 Electrical conductivity.

3 Considers one milking.

4 Calculated for a mastitis case (cow level).

5 Calculated for a mastitis-free milking using only cows that never had mastitis.

6 Based on a model developed for conventional milking and adapted for an automatic milking system.

7 Records with indeterminable (e.g., due to missing values) were excluded.

8 A fuzzy logic was used to classify alerts generated by an earlier developed model [18] in order to decrease the number of false positive alerts, not to increase the sensitivity of the detection model.

9 l-Lactate dehydrogenase.

10 Approximately, using formula: false alert rate ≈ 10 × (100 – specificity) [27].
